# Supplementary material for: Comparative efficacy of gas therapy for diabetic foot ulcers using network meta-analysis
Source: PeerJ. 2025 Jun 16;13:e19571. doi: 10.7717/peerj.19571 (PMC12178245; doi:10.7717/peerj.19571)
Supplement: Supplemental Information 1 [file peerj-13-19571-s001.pdf]

Table S1 1. Complete list of Pubmed electronic library search terms (Updated March 03, 2024)

	Query	Results
#1	diabetic foot[MeSH Terms]	11,816
#2	"diabetic feet"[Title/Abstract] OR "diabetic foot"[Title/Abstract] OR "diabetic foot syndrome"[Title/Abstract] OR "diabetic foot ulcer"[Title/Abstract]	12,456
#3	gas therapy[MeSH Terms]	48,550
#4	"carbon dioxide"[Title/Abstract] OR "carbon dioxide absorption"[Title/Abstract] OR "carbon dioxide formation"[Title/Abstract] OR "carbon dioxide insufflation"[Title/Abstract] OR "carbon dioxide output"[Title/Abstract] OR "carbon dioxide production"[Title/Abstract] OR "carbon dioxide response"[Title/Abstract] OR "carbon dioxide snow"[Title/Abstract] OR "carbon dioxide therapy"[Title/Abstract] OR "carbonic acid gas"[Title/Abstract] OR "carbonic anhydride"[Title/Abstract] OR "carbonic dioxide"[Title/Abstract] OR "carbonic gas"[Title/Abstract] OR "carbonice"[Title/Abstract] OR "CO2"[Title/Abstract] OR "cold atmospheric plasma"[Title/Abstract] OR "dry ice"[Title/Abstract] OR "endogenous nitrate vasodilator"[Title/Abstract] OR "endothelium derived nitric oxide"[Title/Abstract] OR "gas therapy "[Title/Abstract] OR "genosyl"[Title/Abstract] OR "ground level ozone"[Title/Abstract] OR "HBO therapy"[Title/Abstract] OR "high pressure oxygen"[Title/Abstract] OR "high tension O2"[Title/Abstract] OR "high tension oxygen"[Title/Abstract] OR "hyperbaric medicine"[Title/Abstract] OR "hyperbaric O2"[Title/Abstract] OR "hyperbaric oxygen"[Title/Abstract] OR "hyperbaric oxygen therap*"[Title/Abstract] OR "hyperbaric oxygen treatment"[Title/Abstract] OR "hyperbaric oxygenation*"[Title/Abstract] OR "hyperbaric oxygeni?ation"[Title/Abstract] OR "hyperbaric therapy"[Title/Abstract] OR "inomax"[Title/Abstract] OR "kohlendioxyd"[Title/Abstract] OR "kohlensaure"[Title/Abstract] OR "low level ozone"[Title/Abstract] OR "mononitrogen monoxide"[Title/Abstract] OR "nitric oxide"[Title/Abstract] OR "nitrogen monoxide"[Title/Abstract] OR "noxivent"[Title/Abstract] OR "ozon"[Title/Abstract] OR "ozone"[Title/Abstract] OR "topical oxygen therapy"[Title/Abstract] OR "triplet oxygen"[Title/Abstract] OR "tropospheric ozone"[Title/Abstract] OR "ulspira"[Title/Abstract]	393,448
#5	randomized controlled trial[MeSH Terms]	171,068
#6	"randomised controlled study "[Title/Abstract] OR "randomised controlled trial "[Title/Abstract] OR "randomized controlled study "[Title/Abstract] OR "randomized controlled trial"[Title/Abstract]	163,475
#7	(#1 OR #2) AND (#3 OR #4) AND (#5 OR #6)	47

2. Complete list of Embase electronic library search terms (Updated March 03, 2024)

	Query	Results
#1	'diabetic foot'/exp	22,118
#2	'diabetic feet':ti,ab,kw OR 'diabetic foot':ti,ab,kw OR 'diabetic foot syndrome':ti,ab,kw OR 'diabetic foot ulcer':ti,ab,kw	18,261
#3	'gas therapy'/exp	40
#4	'carbon dioxide':ti,ab,kw OR 'carbon dioxide absorption':ti,ab,kw OR 'carbon dioxide formation':ti,ab,kw OR 'carbon dioxide insufflation':ti,ab,kw OR 'carbon dioxide output':ti,ab,kw OR 'carbon dioxide production':ti,ab,kw OR 'carbon dioxide response':ti,ab,kw OR 'carbon dioxide snow':ti,ab,kw OR 'carbon dioxide therapy':ti,ab,kw OR 'carbonic acid gas':ti,ab,kw OR 'carbonic anhydride':ti,ab,kw OR 'carbonic dioxide':ti,ab,kw OR 'carbonic gas':ti,ab,kw OR 'carbonice':ti,ab,kw OR 'co2':ti,ab,kw OR 'cold atmospheric plasma':ti,ab,kw OR 'dry ice':ti,ab,kw OR 'endogenous nitrate vasodilator':ti,ab,kw OR 'endothelium derived nitric oxide':ti,ab,kw OR 'gas therapy':ti,ab,kw OR 'genosyl':ti,ab,kw OR 'ground level ozone':ti,ab,kw OR 'hbo therapy':ti,ab,kw OR 'high pressure oxygen':ti,ab,kw OR 'high tension o2':ti,ab,kw OR 'high tension oxygen':ti,ab,kw OR 'hyperbaric medicine':ti,ab,kw OR 'hyperbaric o2':ti,ab,kw OR 'hyperbaric oxygen':ti,ab,kw OR 'hyperbaric oxygen therap*':ti,ab,kw OR 'hyperbaric oxygen treatment':ti,ab,kw OR 'hyperbaric oxygenation*':ti,ab,kw OR 'hyperbaric oxygeni?ation':ti,ab,kw OR 'hyperbaric therapy':ti,ab,kw OR 'inomax':ti,ab,kw OR 'kohlendioxyd':ti,ab,kw OR 'kohlensaure':ti,ab,kw OR 'low level ozone':ti,ab,kw OR 'mononitrogen monoxide':ti,ab,kw OR 'nitric oxide':ti,ab,kw OR 'nitrogen monoxide':ti,ab,kw OR 'noxivent':ti,ab,kw OR 'ozon':ti,ab,kw OR 'ozone':ti,ab,kw OR 'topical oxygen therapy':ti,ab,kw OR 'triplet oxygen':ti,ab,kw OR 'tropospheric ozone':ti,ab,kw OR 'ulspira':ti,ab,kw	386,660
#5	'randomized controlled trial'/exp	811,985
#6	'randomised controlled study':ti,ab,kw OR 'randomised controlled trial':ti,ab,kw OR 'randomized controlled study':ti,ab,kw OR 'randomized controlled trial':ti,ab,kw	214,898
#7	(#1 OR #2) AND (#3 OR #4) AND (#5 OR #6)	83

3. Complete list of Cochrane Library electronic library search terms (Updated March 03, 2024)

	Query	Results
#1	MeSH descriptor: [Diabetic Foot] explode all trees	1,496
#2	('diabetic feet' OR 'diabetic foot' OR 'diabetic foot syndrome' OR 'diabetic foot ulcer'):ti,ab,kw	4,337
#3	MeSH descriptor: [] explode all trees	0
#4	('carbon dioxide' OR 'carbon dioxide absorption' OR 'carbon dioxide formation' OR 'carbon dioxide insufflation' OR 'carbon dioxide output' OR 'carbon dioxide production' OR 'carbon dioxide response' OR 'carbon dioxide snow' OR 'carbon dioxide therapy' OR 'carbonic acid gas' OR 'carbonic anhydride' OR 'carbonic dioxide' OR 'carbonic gas' OR 'carbonice' OR 'CO2' OR 'cold atmospheric plasma' OR 'dry ice' OR 'endogenous nitrate vasodilator' OR 'endothelium derived nitric oxide' OR 'gas therapy ' OR 'genosyl' OR 'ground level ozone' OR 'HBO therapy' OR 'high pressure oxygen' OR 'high tension O2' OR 'high tension oxygen' OR 'hyperbaric medicine' OR 'hyperbaric O2' OR 'hyperbaric oxygen' OR 'hyperbaric oxygen therap*' OR 'hyperbaric oxygen treatment' OR 'hyperbaric oxygenation*' OR 'hyperbaric oxygeni?ation' OR 'hyperbaric therapy' OR 'inomax' OR 'kohlendioxyd' OR 'kohlensaure' OR 'low level ozone' OR 'mononitrogen monoxide' OR 'nitric oxide' OR 'nitrogen monoxide' OR 'noxivent' OR 'ozon' OR 'ozone' OR 'topical oxygen therapy' OR 'triplet oxygen' OR 'tropospheric ozone' OR 'ulspira'):ti,ab,kw	37,894
#5	MeSH descriptor: [Randomized Controlled Trial] explode all trees	37
#6	('randomised controlled study ' OR 'randomised controlled trial ' OR 'randomized controlled study ' OR 'randomized controlled trial'):ti,ab,kw	834,517
#7	(#1 OR #2) AND (#3 OR #4) AND (#5 OR #6)	125

Table S2 **Mean difference (MD) and 95% confidence interval (CI) for primary outcome measure.** (A) Healing time; (B) Amputation rate; (C) Adverse event.

(A) Healing time

**HBOT**			
-36.02<br>(-122.52, 51.35)	**OT**		
-60.12<br>(-124.99, 4.88)	-24.08<br>(-82.04, 34.15)	**SOC**	
-51.36<br>(-122.22, 19.77)	-15.25<br>(-80.97, 50.23)	8.85<br>(-20.79, 37.58)	**TOT**

(B) Amputation rate

**HBAT**					
-0.10<br>(-0.47, 0.25)	**HBOT**				
**18.96<br>(0.52, 55.34)**	**19.06<br>(0.63, 55.48)**	**NOT**			
0.18<br>(-0.43, 0.83)	0.29<br>(-0.22, 0.83)	**-18.76<br>(-55.13, -0.32)**	**OT**		
**-0.93<br>(-1.40, -0.48)**	**-0.82<br>(-1.12, -0.55)**	**-19.88<br>(-56.31, -1.47)**	**-1.11<br>(-1.59, -0.70)**	**SOC**	
0.90<br>(-0.54, 2.94)	1.00<br>(-0.39, 3.02)	-17.96<br>(-54.48, 0.58)	0.71<br>(-0.72, 2.76)	**1.82<br>(0.47, 3.84)**	**TOT**

(C) Adverse event

**CAP**						
-0.05<br>(-1.60, 1.40)	**HBAT**					
-0.54<br>(-2.00, 0.82)	-0.49<br>(-1.03, 0.02)	**HBOT**				
0.56<br>(-0.59, 1.74)	0.61<br>(-0.67, 2.02)	1.10<br>(-0.06, 2.41)	**NOT**			
-0.81<br>(-3.03, 0.99)	-0.76<br>(-3.04, 1.19)	-0.27<br>(-2.49, 1.62)	-1.37<br>(-3.5, 0.30)	**OOT**		
0.12<br>(-0.83, 1.09)	0.16<br>(-0.92, 1.40)	0.65<br>(-0.29, 1.78)	-0.44<br>(-1.13, 0.21)	0.92<br>(-0.59, 2.96)	**SOC**	
0.44<br>(-0.56, 1.46)	0.48<br>(-0.65, 1.76)	0.97<br>(-0.03, 2.14)	-0.12<br>(-0.88, 0.60)	1.24<br>(-0.31, 3.30)	**0.32<br>(0.01, 0.63)**	**TOT**

Notes.

HBOT, Hyperbaric oxygen therapy; HBAT, Hyperbaric air therapy; TOT, Topical oxygen therapy; OT, Ozone therapy; OOT, Oxygen-Ozone therapy; NOT, Nitric oxide therapy; CAP, Cold atmospheric plasma; SOC, Standard of care.

Statistically significant differences are highlighted in bold.

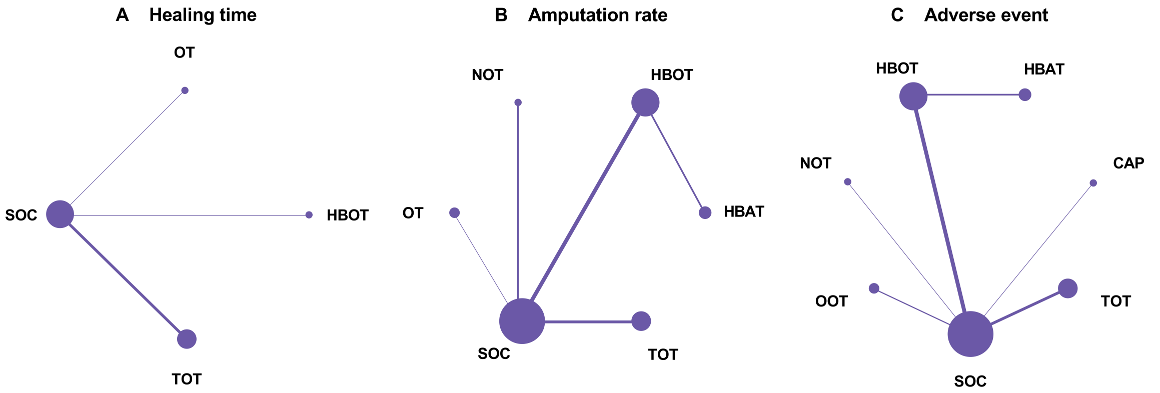

Figure S1 Network plots. (A) Healing time; (B) Amputation rate; (C) Adverse event.

HBOT, Hyperbaric oxygen therapy; HBAT, Hyperbaric air therapy; TOT, Topical oxygen therapy; OT, Ozone therapy; OOT, Oxygen-Ozone therapy; NOT, Nitric oxide therapy; CAP, Cold atmospheric plasma; SOC, Standard of care.

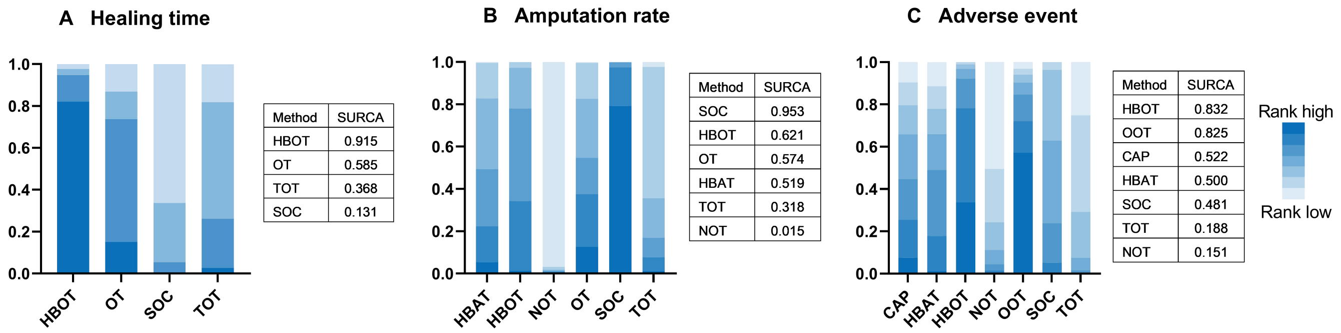

Figure S2 Cumulative probability ranking graph. (A) Healing time; (B) Amputation rate; (C) Adverse event.

HBOT, Hyperbaric oxygen therapy; HBAT, Hyperbaric air therapy; TOT, Topical oxygen therapy; OT, Ozone therapy; OOT, Oxygen-Ozone therapy; NOT, Nitric oxide therapy; CAP, Cold atmospheric plasma; SOC, Standard of care.

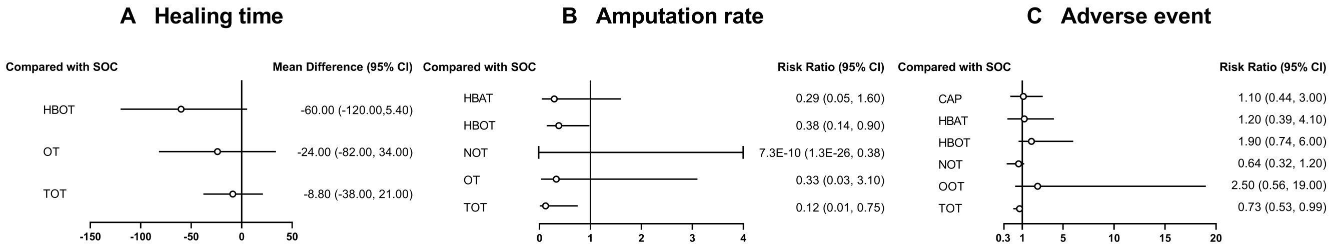

Figure S3 Forest plot of gas therapy compared with SOC. (A) Healing time; (B) Amputation rate; (C) Adverse event.

HBOT, Hyperbaric oxygen therapy; HBAT, Hyperbaric air therapy; TOT, Topical oxygen therapy; OT, Ozone

A Healing rate

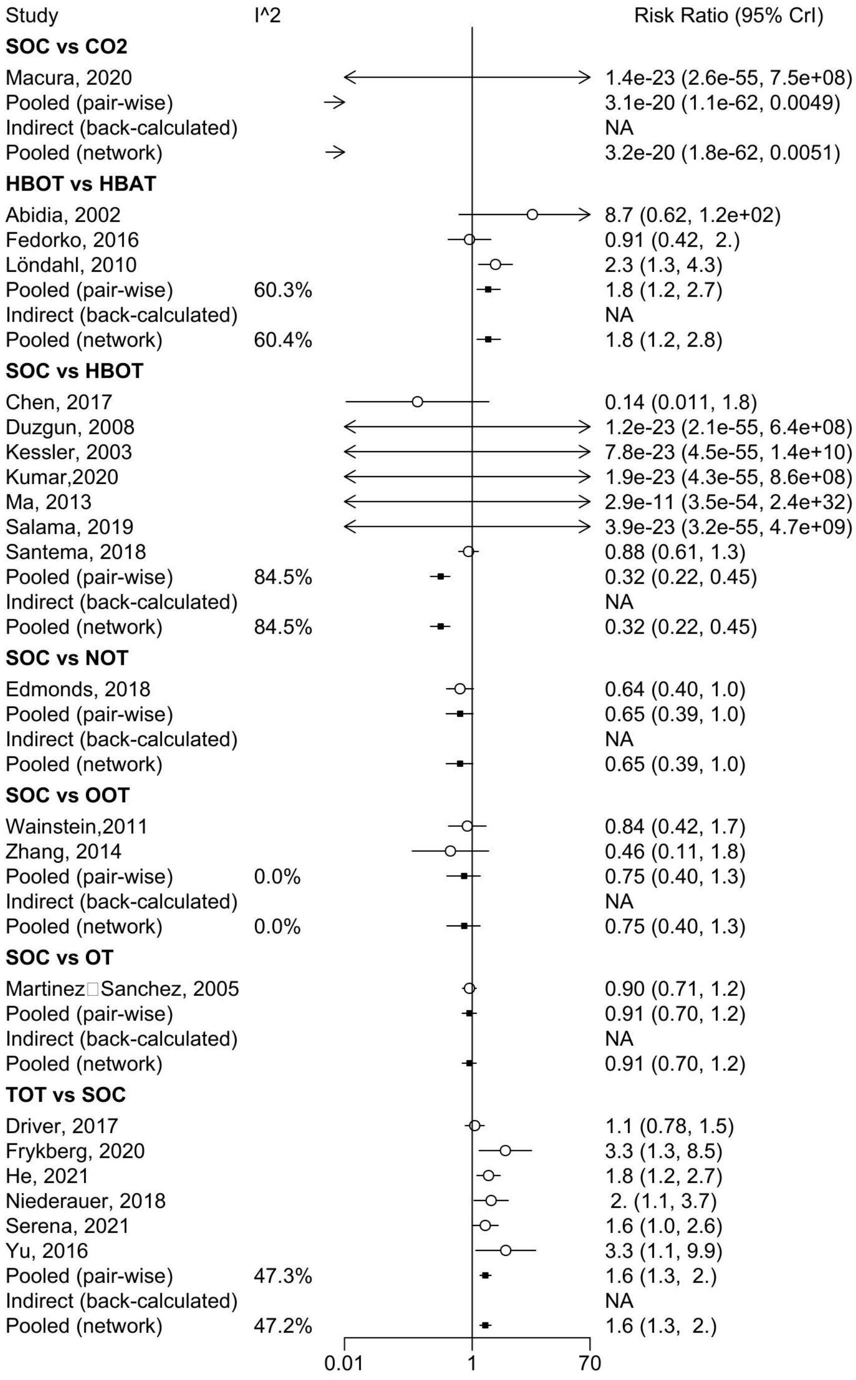

B Area reduction rate

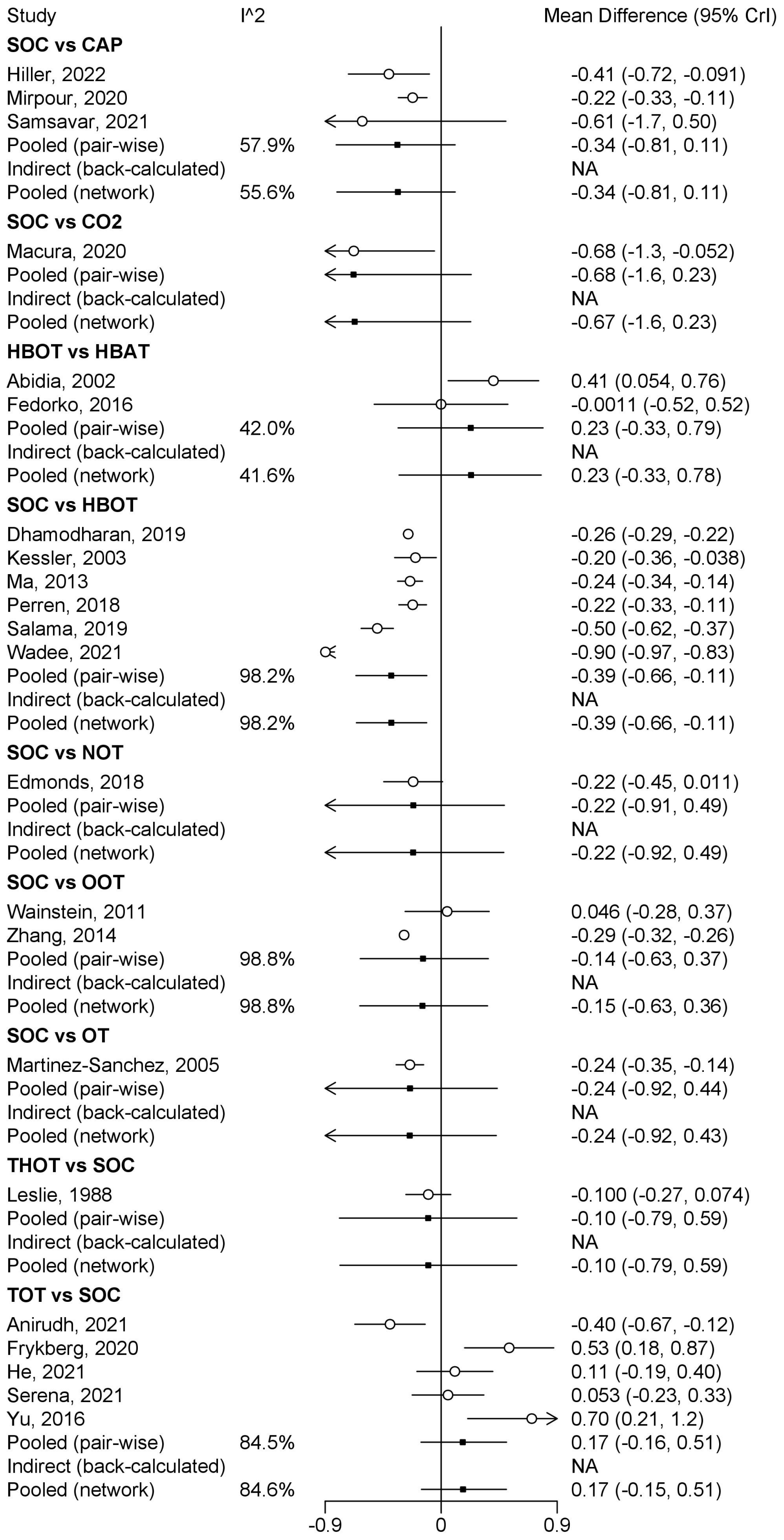

Figure S4 Heterogeneity assessment graph. (A) Healing rate; (B) Area reduction rate.

HBOT, Hyperbaric oxygen therapy; HBAT, Hyperbaric air therapy; TOT, Topical oxygen therapy; THOT, Topical hyperbaric oxygen therapy; OT, Ozone therapy; OOT, Oxygen-Ozone therapy; CO_2, Carbon dioxide; NOT, Nitric oxide therapy; CAP, Cold atmospheric plasma; SOC, Standard of care.
